# Understanding steinstrasse: a systematic review of definitions, clinical management, and emerging perspectives in endourology

**DOI:** 10.1007/s00345-026-06267-7

**Published:** 2026-02-16

**Authors:** F. Zorzi, S. Moretto, L. M. I. Jannello, A. Bravo-Balado, A. Quarà, H. Werth, G. Rossin, A. Alanazi, P. Scilipoti, L. Candela, G. Liguori, S. Doizi, O. Traxer, F. Panthier

**Affiliations:** 1 Sorbonne Université, Service d’Urologie, AP-HP, Hôpital Tenon, Paris, 75020 France; 2 Endolase lab, GRC20-Sorbonne unversity, PIMM-Arts et Métiers Paris Tech, Paris, 75013 France; 3 Urology Unit, Department of Medical, Surgical and Health Sciences, University of Trieste, Trieste, 34127 Italy; 4 Department of Urology, Humanitas Clinical and Research Institute IRCCS, Milan, Italy; 5 Department of Urology, Ospedale Maggiore Policlinico, Foundation IRCCS Ca’ Granda, University, Milano, 20122 Italy; 6 Department of Oncology, Division of Urology, University of Turin, San Luigi Gonzaga Hospital, Orbassano, Italy; 7 Department of Experimental Oncology/Unit of Urology, IRCCS Ospedale San Raffaele, Vita-Salute San Raffaele University, Milan, Italy

**Keywords:** Urolithiasis, Steinstrasse, ESWL, Ureteroscopy, Laser lithotripsy, Endourology

## Abstract

**Introduction:**

To systematically review the definitions, diagnostic modalities, incidence, predictive factors, and management strategies of Steinstrasse (SS), following extracorporeal shock wave lithotripsy (ESWL) and endourological procedures.

**Methods:**

A comprehensive literature search was conducted in accordance with the PRISMA guidelines across PubMed, Scopus, and Embase, identifying randomized controlled trials, prospective and retrospective observational studies, and case series published between 1986 and May 2025.

**Results:**

Forty-two studies met the inclusion criteria. The reported incidence of SS varied ranged from 1% to 23%, depending on initial stone burden, location, and treatment modality. Historical classifications, such as that proposed by Coptcoat et al., remain the most adopted linking SS morphology to clinical management. Diagnostic methods have evolved over time, from plain abdominal radiography (KUB) to non-contrast computed tomography (NCCT). Conservative management achieved spontaneous clearance in nearly half of reported cases, whereas ureterorenoscopy represents the treatment of choice in the remaining persistent cases. Recent technological advances in flexible Ureterorenoscopy (FURS), laser systems, and the use of flexible aspirating navigable ureteral access sheaths (FANS) have drawn attention to the accumulation of fine residual fragments (≤ 4 mm) and dust, potentially leading to SS-like conditions.

**Conclusions:**

Although SS has been traditionally associated with ESWL, its occurrence following FURS is an emerging concern. The present review highlights the importance of early recognition, standardized definitions, and individualized management strategies to optimize outcomes in both ESWL- and endoscopy-related SS.

**Supplementary Information:**

The online version contains supplementary material available at 10.1007/s00345-026-06267-7.

## Introduction

The global incidence of urolithiasis has risen markedly over the past decade, driven by metabolic disorders, environmental factors, and an aging population [1–3]. Current estimates indicate that the prevalence of kidney stones ranges between 5% and 10% in both the United States and Europe. Moreover, up to 50% of patients with urinary stone disease are expected to experience at least one recurrent episode during their lifetime [4]. Consequently, urolithiasis represents a major source of morbidity and a growing healthcare burden worldwide.

Interventional options for the treatment of urinary stones include flexible ureteroscopy (FURS), percutaneous nephrolithotomy (PCNL), and extracorporeal shock wave lithotripsy (ESWL) [5]. FURS and PCNL are minimally invasive surgical approaches that typically require anaesthesia, whereas ESWL remains the only non-invasive therapeutic modality for urinary stone treatment, apart from conservative management and expulsive therapy [6].

The success of ESWL depends on complete stone clearance, which in turn relies on the fragmentation and spontaneous passage of fragmented calculi through the urinary tract [7, 8]. One of the most concerning complications of ESWL is obstructive uropathy caused by acute ureteral obstruction due to stone fragments [9]. When multiple fragments align and obstruct the ureter, this condition is termed Steinstrasse (SS) [10]. Despite continuous technological improvements in shock wave lithotripters, SS remains a clinically significant challenge, as it may lead to obstructive uropathy, renal function loss, pain, superimposed infection, or even urosepsis.

In recent decades, significant technological innovation has been directed toward improving stone-free rates (SFRs) through enhanced endoscopic performance. Advances such as the development of flexible aspirating navigable ureteral access sheaths (FANS) and aspirating flexible instruments have expanded the indications for FURS, enabling the effective treatment of increasingly larger stone burdens [11]. Concurrently, the widespread adoption of novel laser systems—particularly the thulium fiber laser (TFL) and pulsed thulium: yttrium–aluminium–garnet (p-Tm: YAG) laser—has facilitated efficient “dusting” lithotripsy [12]. The interaction between these energy sources and calculi produces large quantities of fine stone fragments and dust. However, if these particles are retained within the ureter, they may lead to partial obstruction or SS-like conditions [13].

A major limitation in the existing literature on SS is the absence of a universally accepted definition, resulting in substantial variability in reported incidence, diagnostic criteria, and management approaches. This systematic review aims to clarify these inconsistencies by summarizing existing definitions and evaluating their clinical implications across both endoscopic and extracorporeal lithotripsy procedures.

## Methods

### Research strategy

This systematic review was conducted according to the principles outlined by the European Association of Urology (EAU) Guidelines Office and the Preferred Reporting Items for Systematic Reviews and Meta-analyses (PRISMA) recommendation [14, 15]. The protocol was registered in the International Prospective Register of Ongoing Systematic Reviews (PROSPERO) database (registration number CRD42024621052). A comprehensive electronic literature search was conducted using the Scopus, Embase, and Medline/PubMed up to 31 May 2025. The search strategy followed predefined inclusion criteria and combined the following terms using Boolean operators: (“steinstrasse” OR “sandstrasse” OR “stein street” OR “stone street”) AND (“urolithiasis” OR “kidney stones” OR “renal calculi” OR “ureteral calculi” OR “urinary calculi” OR “stones” OR “calculi”). A detailed overview of the search strategy is provided in the Supplementary Materials (Appendix 1).

### Inclusion and exclusion criteria

Study selection was performed according to the PICOS framework: Population (P), Interventions (I), Comparator (C), Outcome (O), and Study design (S). They were set as follows: Population—patients presenting SS; Intervention—ESWL or endourological procedures, including PCNL and retrograde ureteroscopy (URS); Comparator— none; Outcome—assessment of the definition of SS, its relationship with urolithiasis treatments, the methods for diagnosis and management; and Study—prospective studies (RCTs and prospective observational studies) and retrospective studies (Observational studies).

No restriction was applied to the number of patients included in the studies. The initial screening was independently performed by three investigators (F.Z., A.Q., G. R.) based on the titles and abstracts of the article to identify potentially eligible reports. Only English-language articles were assessed for inclusion. No restriction on the publication date was applied. Reviews, meta-analyses, editorials, commentaries, authors’ replies, meeting abstracts of unpublished studies, and case reports were excluded. The reference section of each article was checked to avoid omitting relevant articles. Potentially relevant studies were subjected to a full-text review, and the relevance of the reports was confirmed after the data extraction process. Duplicate studies from the same research groups were excluded, retaining the most recent fulfilling the selection criteria and the most recent ones; however, if they fulfilled the selection criteria and provided additional information on outcomes of interest, they were retained for these outcomes only. Disagreements were resolved by consultation with the senior co-authors (S.D., O.T., F.P.).

### Data extraction

Data on study, patient, and treatment characteristics were independently extracted by three authors (H.W., L.M.I.J. and A.B.). The following variables were collected from the included studies: first author, year of publication, study design, number of patients, definition and incidence of SS, stone characteristics (location, size, Hounsfield Units), treatment modalities (semirigid ureteroscopy, FURS, re-ESWL), treatment parameters (ESWL energy and frequency, number of shock waves, number of eswl sessions, type of lithtotripter used, PCNL energy used, FURS type of energy applied and laser settings), imaging modality used for diagnosis, SS length and location, reported outcomes such as SS clearance, SFR, and complications related to SS management.

### Risk of bias (RoB) assessment

Two reviewers (S.M. and S.D.) independently assessed the methodological quality of included studies. The risk of bias (RoB) for non-randomized studies was assessed using the ROBINS-I tool [16], with each domain and the overall risk categorized as “low,” “moderate,” “serious,” or “critical”. For Randomized Controlled Trials (RCTs), the RoB was evaluated using the ROBINS-II tool [17], and classified as ‘‘low’’, ‘‘some concerns”, or ‘‘high’’. In the case of prospective and retrospective single-arm studies, RoB assessment followed the European Association of Urology (EAU) guidelines for systematic reviews of case series, with studies judged as having either “low” or “high” risk. Potential confounders were identified by consensus between the two reviewers based on the existing literature. Any discrepancies were resolved through discussion with senior co-authors (S.D., O.T., F.P.).

## Evidence synthesis

### Study selection and characteristics

**Fig. 1:**
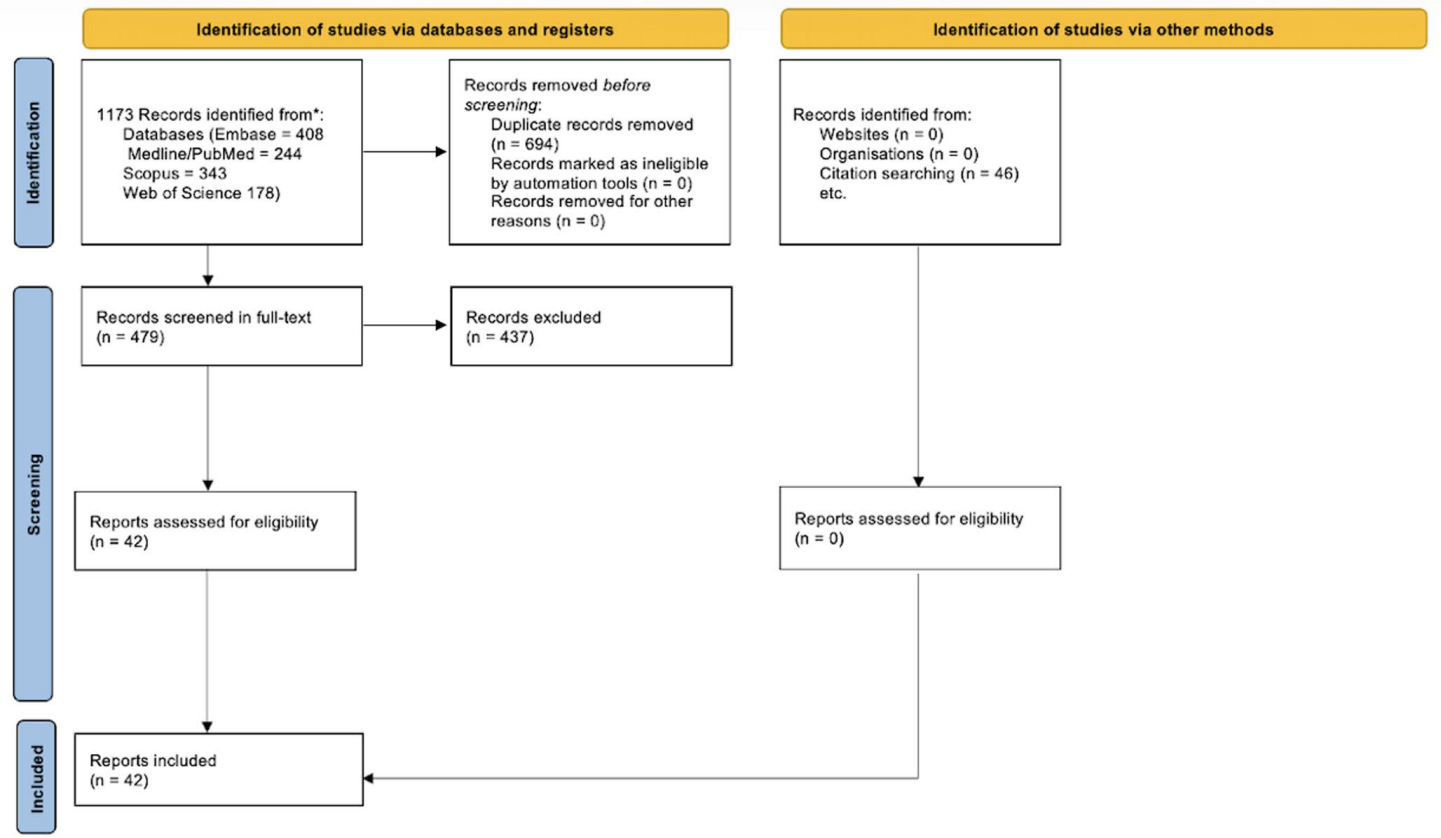
PRISMA flowchart

The study selection process is illustrated in the PRISMA flowchart (Fig. 1). A total of 1,173 records were initially identified. After screening, 479 full-text articles were assessed for eligibility, of which 42 studies met the inclusion criteria. These comprised RCTs, non-RCTs, single-arm case series published between 1986 and 2025. The remaining 437 full-text articles were excluded as out of scope.

### Risk of bias

Quality and RoB assessments are summarized in Supplementary Table 1 (RCTs), Supplementary Table 2 (non-randomized studies of interventions), and Supplementary Table 3 (single-arm studies). Among RCTs, RoB assessment identified at least some concerns in 7 of 8 RCTs, while only one trial judged to be at low risk of bias. Among non-randomized studies, 4 out of 9 were considered to have low RoB, whereas the remaining 5 were judged to have a moderate RoB. Most single-arm studies (20/25) were classified as having high RoB, primarily because of limited standardization of SS definitions and incomplete outcome reporting.

### Definition and historical perspectives of Steinstrasse

Table 1 Historical definitions of the SS phenomenon after ESWL, including study characteristics, SS definitions, incidence rates, management approaches, and outcomes.

**Table 1:** Historical definitions of the SS phenomenon after ESWL, including study characteristics, SS definitions, incidence rates, management approaches, and outcomes

Authors	Study design/ Population	Study aim	Definition of SS	Incidence of the SS	SS management	Outcomes and complications
Coptcoat M (1986)	Retrospective, Adults (*n* = 600)	To analyze complications occurred after SWL treatment	Defined as natural phenomenon where stone particles move collectively down the ureter following ESWL	Complicated SS occurred in 32 cases (5%)	Fragments located in the upper ureter removed by PCNL; stones in the lower ureter managed via primary PNS positioning, URS, or ureterolithotomy	Incidence of complicated steinstrasse was directly related to the initial stone size of stones treated by ESWL alone
Coptcoat M (1988)	Prospective Observational, Adults (*n* = 600)	To evaluate SS management	Classified of SS into three categories (Fig. 2): • Type I: Fragments ≤ 2 mm • Type II: Leading large fragment (4–5 mm) with a tail of smaller fragments ≤2 mm • Type III: Large retained fragments	32 SS cases (mean length of 4.5 cm); • 17cases type I; • 11 cases type II; • 4 cases type III	Management based on three clinical groups (Table 2b): • Group 1: asymptomatic (observation) • Group 2: Symptomatic cases without proximal ureteral dilatation (antibiotics, observation) • Group 3: Symptomatic cases with proximal dilatation or fever (immediate PNS)	Among 24 patients treated with preventive PNS, 18 passed stones spontaneously, 5 required URS, and 1 underwent ureterolithotomy; additionally, 5 underwent primary URS and 3 PCNL
Weinerth J L et al. (1989)	Retrospective, Adults (*n* = 650)	To evaluate the management of large SS (> one-third ureteral length)	Defined as the development of multiple stone fragments within the ureter after ESWL	19 out of 650 patients (2.9%) presented large SS. 6 of them had both ureters involved.	In acute cases immediate PNS diversion; Non acute patients managed through either expectant management, URS ultrasonic lithotripsy, or laser lithotripsy.	Most commonly located in the distal third of the ureter; SS length 9–17 cm, fragment size 2–5 mm
Böhle et al. (1989)	Retrospective, children (*n* = 24)	To evaluate long-term ESWL effects in pediatric kidneys	SS named as urinary flow obstruction due to ureteral calculi after ESWL treatment	2 out of 24 (8.3%)	PNS placement in both patients to preserve renal function	SS resolved spontaneously within five days post-PNS
Grasso et al. (1995)	Prospective observational, Adults (*n* = 121)	To identify factors associated with ESWL failure in upper-tract calculi	Defined as multiple stone fragments distributed along the ureter, often with residual renal calculi	10 out of 121 (8.26%); mean pre-treatment mean stone diameter 33.7 mm	Combined retrograde or antegrade URS lithotripsy and PCN with ultrasonic lithotripsy	Delayed treatment of complicated SS associated with irreversible renal deterioration and ureteral stricture formation

*SS* Steinstrasse, *ESWL* extracorporeal shock-wave lithotripsy, *URS* retrograde ureterorenoscopy, *PCNL* percutaneous nephrolithotomy, *PNS* percutaneous nephrostomy

SS - a German term meaning “stone street” - was first described in 1986 as a post-ESWL phenomenon in which fragmented stone particles align within the ureter, potentially leading to urinary obstruction [18] (Table 1). The “Complicated” form of SS was initially defined as the accumulation of stone fragments causing obstruction, loin pain, progressive proximal ureteral dilatation, with or without superimposed infection. In such cases, decompression of the collecting system by percutaneous nephrostomy (PNS) is mandatory to relieve obstruction and control infection. If fragments are not spontaneously cleared by the urinary system, semirigid and FURS — rarely PCNL and anterograde FURS — recommended to ensure complete fragment clearance.

**Fig. 2:**
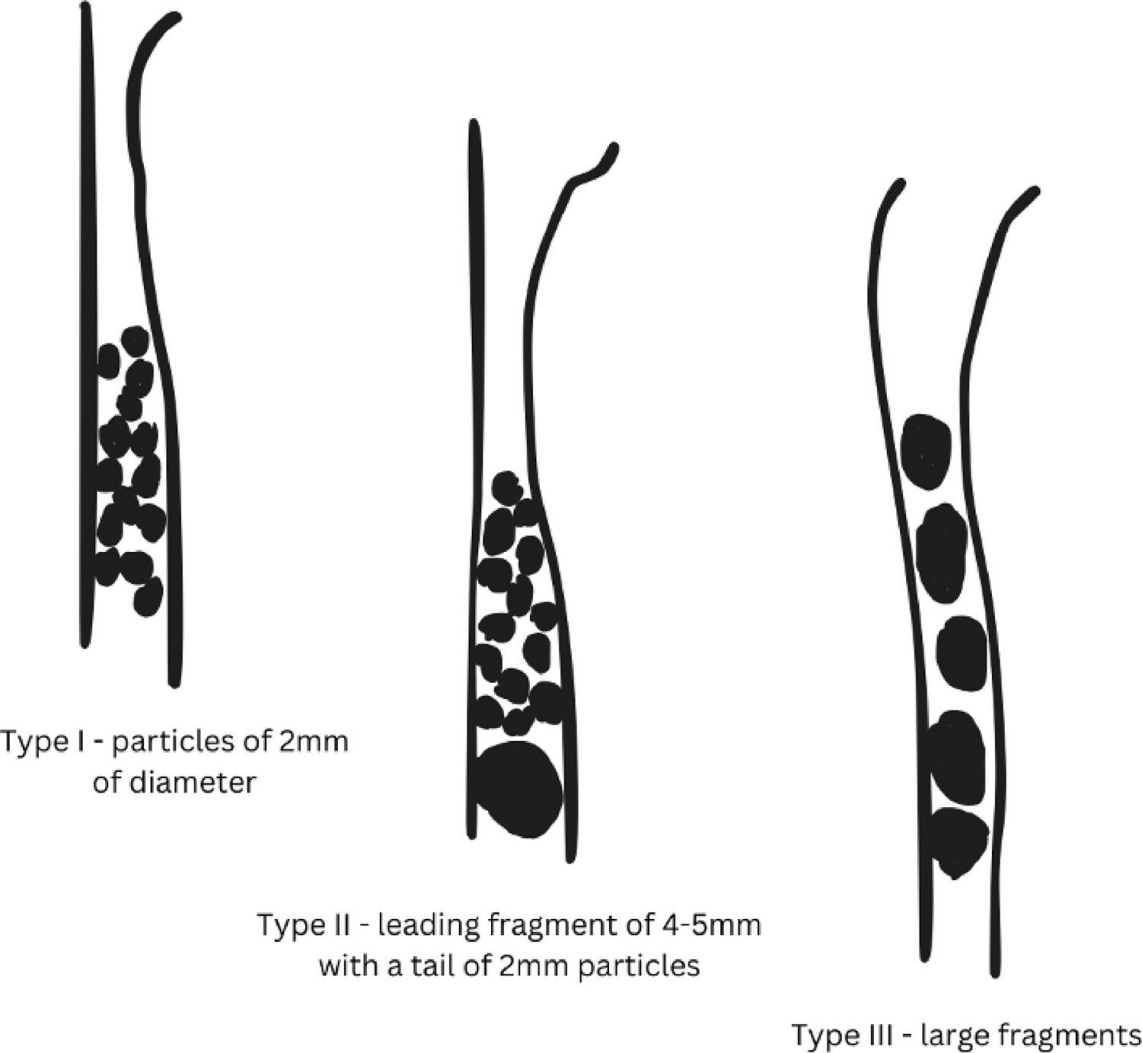
Schematic representation of SS stages based on the classification proposed by Coptcoat et al. (1988)

Coptcoat et al. subsequently proposed a classification system dividing SS into three groups according to presentation and obstruction severity (Fig. 2.) [19]. Group 1 included asymptomatic patients managed expectantly to allow spontaneous passage of fragments. Group 2 included patients presenting with colicky pain without proximal ureteral dilatation, in whom antibiotic therapy was recommended to allow safe observation. Group 3 included symptomatic patients presenting with hydronephrosis or fever, in whom immediate PNS placement was deemed mandatory to prevent progression to urosepsis. In this latter group, definitive fragment clearance was delayed and performed endoscopically approximately three weeks later (Supplementary Table 4. ,* categorized according to clinical presentation*,* imaging findings*,* and recommended therapeutic strategy*). Although URS achieved the highest success rate in this series, the authors advised a combined endoscopic approach or open ureterolithotomy in case of treatment failure. Similar management strategies were later successfully adopted in paediatric population.

In 1989, Böhle et al. were the first to report SS in children. They described a series of 24 children treated with ESWL for upper urinary tract stones, two of them developed ureteral obstruction that resolved spontaneously within five days after PNS placement [20].

Winerth et al. observed SS 19 out of 650 patients (2,9%), characterized by fragment columns measuring from 9 to 17 cm and extending over more than one-third of the ureteral length [21]. Plain abdominal radiography (KUB) imaging revealed the ureterovesical junction was the most affected site.

Grasso et al. defined the SS as the aggregation of multiple stone fragments along the ureter, frequently associated with residual renal calculi [22]. In their prospective series of 121 patients referred after ESWL, SS occurred in 10 cases (8.3%) with a mean pre-treatment stone burden of 33.7 mm. The authors emphasized that delayed intervention for complicated SS with large fragments and prolonged observation could result in irreversible renal impairment and ureteral stricture formation.

### Incidence, diagnostic methods, and clinical insights

Table 2. Studies providing diagnostic insights into SS following ESWL: lithotripter type, imaging applied used for diagnosis, SS characteristics, incidence, and proposed treatments.

**Table 2:** Studies providing diagnostic insights into SS following ESWL: lithotripter type, imaging modalities applied for diagnosis, SS characteristics, incidence, and proposed treatments

Authors (year)	Study design	Number of patients	Study aim	Lithotripter type/ treatment settings	Definition of Steinstrasse	Imaging modality used for diagnosis	Steinstrasse localization and dimensions	Incidence of Steinstrasse	Treatment approach
Madbouly et al. (2002)	Retrospective, Adults	4634	To evaluate predictive factors of SS formation	Electrohydraulic; Energy delivered between either 14–17 kV, 18–22 kV, or greater 22 kV	Retained ureteral stones causing partial or complete ureteral obstruction	KUB	74% SS localized in pelvic ureter, 21.7% in lumbar ureter, 4.3% iliac ureter; SS length < 1 cm in 53,8%, 1–3 cm in 24.5%, > 3 cm in 21.7% of cases.	SS recorded in 184 patients (3.97%); 74 patients required intervention.	In 110 (59,8%) efficacy of expectant management for fragment clearance; In 74 (40,2%) cases intervention was required: primary URS in 64, PNS in 4, DJ in 5, open ureterolithotomy in 1 case.
Soyupek S. et al. (2005)	Retrospective, Adults	563	To evaluate the stone- and therapy-related factors influencing SS formation	Electrohydraulic; Energy delivered between 9 to 23 kV	Accumulation of stone fragments obstructing the ureter after SWL	ND	84.3% in pelvic ureter, 7.84% in iliac ureter, 7.84% in lumbar ureter	46 out of 563 (8.17%)	ND
Yoshida S. et al. (2007)	Retrospective, Adults	53	to evaluate whether three-dimensional NCCT assessment could predict SS formation in stones < 20 mm	Electromagnetic; Energy delivered up to 3500 shockwaves per treatment	Fragments of stone forming a column (street of stone) longer than 25 mm on KUB film	KUB, NCCT	28.6% in the upper ureter, 71.4% in lower ureter	7 out of 53 (13.21%)	ND
Shrestha B. et al. (2010)	Prospective observational, Adults and children	100	To evaluate ESWL efficacy and factors which might influence the outcome and adverse effects of ESWL	Electrohydraulic; Energy between 10–22 kV, up to 4000 shocks per session	Accumulation of stone fragments within the ureter after SWL stone fragmentation	KUB, US	93.6% in lower ureter	47 (47%) patients developed SS	Expectant management, ancillary procedures (not specified)
Lucio J. II et al. (2011)	Prospective observational, Adults	175	To evaluate factors and outcomes associated with SS after ESWL	Electromagnetic; Shock wave frequency 4000 for renal and 5000 for ureteral calculi.	Presence of more than one ureteral stone simultaneously	KUB, US, CT	42.9% in mid ureter, 35.7% in lower ureter, 21.4% in upper ureter	SS occurred in 14 procedures (5.3%)	MET and expectant management, URS, ESWL targeting SS
Al-Marhoon et al. (2013)	Retrospective, Adults	225	To assess the safety and efficacy of SWL for management of renal and ureteral stone	Frequency between 3000–3500 shocks for renal stones; 3500–4500 for ureteral stones	Complication of SWL in which stone fragments block the ureter to form a stone street	KUB, US	ND	7 (3.1%) out of 225	Expectant management, URS, ESWL targeting SS
El-Assmy et al. (2015)	Retrospective, Children	317	To evaluate how stone- renal- and therapy factors could affect SS formation after ESWL	Electromagnetic; ESWL performed between 12–16 kV, frequency at 80 shocks/min and 2000 shocks/session	Presence of more than one ipsilateral ureteral stone simultaneously after SWL	KUB, US, excretory urography, NCCT	74.1% pelvic ureter, 18.5% lumbar ureter; 7.4% iliac ureter	Overall incidence of SS 27 out of 317 (8.5%)	Expectant management, ESWL targeting SS, URS, PNS in clinically symptomatic obstructions
Phukan et al. (2016)	Retrospective, Adults	2436	To determine the factors that predict the need for intervention in patients with SS	Energy delivered between 8 and 14 kV for renal calculi, 16 kV for ureteric calculi; 1500 shocks per treatment for renal stones; 2000 for ureteric stones	Aggregation of stone fragments within the ureter following ESWL	KUB	77% distal ureter, 17% proximal ureter, 6% mid ureter	89 (3.6%) out of 2436	Expectant management, ESWL on SS, PCN, URS, double J stenting in clinically symptomatic obstructions
Kang et al. (2018)	Retrospective, Adults	551	To identify predictive factors and clinical course of SS formation following SWL for ureteral stone	Electroconductive or electromagnetic; shocks interval between 2500 and 4000 per session	Finely disintegrated stone particles forming a column of sand-like fragments obstructing the ureter	NCCT	100% located in the upper ureter	12 (2.18%) out of 551	Expectant management, ESWL on SS fragments
Dobrowiecka et al. (2018)	Retrospective, Children	247	To analyse the frequency and type of early complications of SWL	Electromagnetic; Energy range between 10-19 kV, overall pulses between 1500–3000 per treatment	Urinary obstruction caused by the partial blockage of the ureter by stone fragments	ND	ND	25 (10.73%) out of 247	URS, DJ stenting in clinically symptomatic obstructions
Pricop et al. (2022)	Retrospective, Adults	106	To determine differences in efficacy and safety in the use of electrohydraulic vs. electromagnetic lithotripters for single kidney stone from 10 to 20 mm	Electrohydraulic and electromagnetic lithotripter; ND	Accumulation of obstructive lithiasis fragments following SWL, sometimes asymptomatic or transient.	KUB	ND	7 (6.6%) out of 106	ESWL targeted on distal SS fragment
Alobaidy et al. (2022)	Single-blinded RCT, Adults	96	To compare grooved and smooth ureteral DJ stent in humans pre-SWL	ND	Column of stone fragments that forms and blocks the ureter	ND	ND	Grooved DJ stents showed higher stone clearance compared with smooth stents	Grooved DJ stent were associated with a higher post-operative urinary tract infection rate

*SS* Steinstrasse, *ESWL* extracorporeal shock wave lithotripsy, *URS* Ureterorenoscopy, *PNS* percutaneous nephrostomy, *NCCT* non-contrast computed tomography, *KUB*, plain radiography, *US* ultrasound, *CT* computed tomography, *SFR* stone-free rate

Initially, patients with SS were categorized as *uncomplicated* or *complicated* according to the need for urgent intervention. Over time, however, the term has been broadened to encompass any ureteral obstruction induced by stone fragmented by ESWL (Table 2). SS is now recognized to results from multiple retained stone fragments obstructing the ureteral lumen after treatment [23–26]. Pricop et al. demonstrated that SS incidence did not significantly differ between electrohydraulic and the electromagnetic lithotripters [27].

Parmar et al. identified 28 cases (4.1%) of SS among 684 patients undergoing ESWL for renal and ureteral stones. Notably, nine patients presented with spontaneous SS without any prior history of ESWL, highlighting that SS can rarely occur in the absence of lithotripsy and supporting the importance of metabolic evaluation such as hypercalciuria, hypocitraturia, and renal tubular acidosis [28].

Advances in imaging have profoundly influenced both the definition and diagnosis of SS. KUB was originally the standard post-ESWL tool; however, its limited accuracy in assessing fragment size and its inability to determine the severity of obstruction restrict its diagnostic performance. The advent of non-contrast-enhanced computer tomography (NCCT) provided superior visualization and precision [29]. Yoshida et al. evaluated the predictive value of helical NCCT, defining SS as a column of stone fragments exceeding 25 mm in length on KUB, and suggested that attenuation values above 650 Hounsfield units (HU) could predict SS formation [30].

Furthermore, Kang et al., in a series of 1,418 patients who underwent single-session ESWL, identified low-dose NCCT parameters predicting SS formation [25]. In particular, larger stone size, higher stone attenuation, and shorter skin-to-stone distance (SSD) were associated with SS development. Therefore, low-dose NCCT represents the preferred imaging modality for diagnosing SS, and stone parameters can serve as predictive factors of SS development after ESWL.

It is plausible that, in addition to discrete fragments > 5 mm, fine debris produced by ESWL may aggregate with small particles within the ureter, contributing to temporary obstruction even in the absence of a dominant leading fragment [31].

In long-term follow-up, high-resolution ultrasonography (US) may serve as an alternative, enabling detection of SS and associated hydronephrosis while avoiding radiation exposure.

### Relationship between Steinstrasse and endourological treatments

Since the introduction of ESWL, SS has been most frequently observed in patients with high stone burden (Supplementary table 5). In this context, ureteral double-J (DJ) stenting has been discussed as a potential means to facilitate fragment clearance and reduce ureteral obstruction; however, the available evidence is mainly mechanistic and does not support routine prophylactic stenting [32] .

Anderson et al. evaluated 1,000 ESWL procedures, including 41 patients with renal stones larger than 3 cm. They compared outcomes among patients treated with ESWL alone, ESWL for residual calculi after PCNL, and ESWL with ureteral stenting [33]. They reported a significantly higher incidence of SS among patients treated with ESWL alone. These cases were typically managed with urgent or emergent DJ stenting, whereas uncomplicated presentations were treated conservatively or with endoscopic approaches. Persistence of SS at three-month follow-up was notably higher in patients treated solely with ESWL for stone burdens greater than 30 mm.

Fine et al. observed that ureteral stents not only prevented urinary obstruction but also facilitated faster dispersion and passage of fragments after ESWL. They hypothesized that stents could mitigate blockage caused by fine debris generated from large-stones [32].

Alobaidy et al. compared grooved versus smooth DJ stents placed before ESWL and found that grooved stents promoted superior fragment clearance [34]. A threshold stone size of 21 mm has been proposed as the upper limit for safe ESWL monotherapy to minimize SS risk [35]. Sayed et al. advocated conservative management as the most appropriate initial strategy, reporting spontaneous resolution in nearly half of SS cases [10]. Predictive factors significantly associated with SS formation included stone size and location, pre-treatment collecting-system dilatation, and ESWL energy settings [36].

Although SS is classically linked to ESWL, it has also been documented following endourological procedures. Ivanov et al. reported one case of SS among 76 patients treated with Holmium: yttrium-aluminium-garnet (Ho: YAG) laser lithotripsy using a disposable ureteroscope; this case was successfully managed with second-look ureteroscopy [37]. These findings broaden the concept of ureteral obstruction after endoscopic procedures, now encompassing both retained fragments and fine particulate “renal sand” [32].

### Efficacy of surgical endourological treatments for Steinstrasse

Table 3. Outcomes and complications of endourological interventions proposed for SS clearance, including lithotripsy settings, stone-fragment characteristics, SFRs, and complication profiles.

**Table 3:** Outcomes and complications of endourological interventions proposed for the clearance of SS, including lithotripsy settings, stone-fragment characteristics, SFRs, and complication profiles

Authors, year	Study design	Number of patients	Study aim	Endourological procedure/lithotripsy settings	Steinstrasse definition	SS location	Treatment outcomes	Treatment complications
Chaussy et al. (1987)	Retrospective	118	To evaluate ultrasonic URS lithotripsy utilizing a solid-wire probe	URS; ultrasonic lithotripsy with 2.7 F solid-wire probe	Retained ureteral fragments following ESWL	Upper ureter 16 (13.5%), mid 13 (11%), lower 89 (75.4%)	SS treated in 38 (32%) cases; total success rate 96.6% (upper 87.5%, mid 96.9%, lower 98.8%)	One ureteral stricture required reimplantation; two cases of post-operative fever, one case of NFS positionement for ureteral oedema
Rabbani et al. (2008)	Retrospective	76	To evaluate URS lithotripsy for SS after ESWL	URS lithotripsy with 8–9.8 F semirigid scope	Fragments column occupying more than 17% of the length of the ureter	Upper 20.8%, mid 8.3%, lower 70.8%;	SS clearance in 14/24 (58.3%); 6 partial responses treated with further URS or ESWL; 4 failed cases managed by open ureterolithotomy. According to Coptcoat classification of SS, success rate was 6/8 for type 1, 8/12 for type 2, and 0/4 for type 3 SS.	One non-functional kidney; no major peri-operative complications reported
Feng et al. (2013)	Retrospective	21	To evaluate Ho: YAG laser lithotripsy for ureteral SS ESWL	Semirigid URS; Ho: YAG lithotripsy	Severe ureteral obstruction with renal function impairment	Upper 7 (33.3%), mid 3 (14.3%), lower 11 (52.4%)	Success 19/21 (90.48%)	1 post-ESWL for retropulsion, 1 open surgery due to ureteral kinking
Wang et al. (2021)	Prospective observational	35	To assess efficacy of VAS for complex SS after ESWL, PCNL or RIRS	Semirigid URS (7/8.4 F) with 12/14Fr VAS; Ho: YAG laser 25–30 Hz, 0.5–0.6 J	Complex SS: ≥ 4 fragments or aggregate length ≥ 1.5 cm	ND	100% SS clearance	ND
Yuming et al. (2024)	Retrospective Observational	31	To evaluate rigid URS with pressure-controlling ureteral access sheath for complex SS	Pressure-controlling access sheath, semirigid URS, Ho: YAG 20–30 Hz and 2–2.5 J	Complex SS: ≥ 4 stones or aggregate length ≥ 1.5 cm	Upper 13, mid 9, lower 5, mixed 4	SFR 96.6% at 1 month	No Clavien–Dindo grade ≥ II complications

*SS* Steinstrasse, *ESWL* extracorporeal shock wave lithotripsy, *URS* ureterorenoscopy, *PCNL*, Percutaneous Nephrolithotomy, *PNS* percutaneous nephrostomy, *NCCT* non-contrast computed tomography, *KUB* plain radiography, *US* ultrasound, *VAS* vacuum-assisted access sheath, *DJ* Double-J stent, *Ho: YAG = Holmium*: Yttrium–Aluminium–Garnet laser, *CT* computed tomography, *SFR* stone-free rate

Since the original description by Chaussy et al. (1987), URS lithotripsy has been regarded as the primary therapeutic approach for SS management [38]. However, its efficacy varies widely according to the technique and technology employed (Table 3).

Rabbani et al. defined the SS as a column of fragmented calculi occupying more than 17% of the ureteral length. In 24 patients treated with URS lithotripsy, a complete SFR after initial procedure was achieved in 14 (58.3%). Six patients (25%) showed partial response, requiring additional ESWL or URS, while four (16.7%) underwent open pyelolithotomy owing to treatment failure [39].

Conversely, Feng et al. evaluated Ho: YAG lithotripsy for severe SS with marked ureteral obstruction and renal functional impairment, achieving success in 19 of 21 patients (90.5%). Of the two failures, one required secondary ESWL due to fragments retropulsion, and the other underwent open surgery because URS was precluded by a kinked upper ureter [40].

Recent innovations aim to minimize the invasiveness of ureteral lithotripsy. Wang et al. reported successful SS clearance using semirigid URS combined with a 12/14 Fr suction ureteral access sheath [41]. Likewise, Yuming et al. described URS as the optimal treatment for significant ureteral obstruction caused by large volumes of crushed stone following endoscopic procedures [42]. They defined complex SS as the presence of four or more stones, or an aggregate fragment length ≥ 1.5 cm. Using Ho: YAG lithotripsy and a novel pressure-controlled access sheath, they achieved a 96.6% SFR at one-month follow-up.

### Medical expulsive therapies for SS clearance

A pivotal role in SS management is played by medical expulsive therapy (MET), typically consisting of an α-blocker combined with non-steroidal anti-inflammatory drugs (NSAIDs) and serial imaging follow-up [33, 35, 36, 43, 44](Supplementary table 6). α-blockers are thought to facilitate the passage of stone-fragments by relaxing ureteral smooth muscle and reducing intraluminal resistance. Their precise efficacy, however, remains debated.

Resim et al. demonstrated in an RCT that α-blockers therapy significantly increased fragment clearance and reduced SS incidence after ESWL compared with analgesics alone [45]. Conversely, other authors have questioned its preventive value [46]. In the RCT by Moursy et al. reported that α-blocker administration did not significantly enhance SS clearance compared with analgesics alone; however, it halved the incidence of SS and reduced NSAID consumption [47]. These findings were confirmed by Ahmed et al., who further demonstrated a reduced need for adjuvant interventions [48].

Additional pharmacological strategies, such as combining phosphodiesterase-5 inhibitors with α-blockers, have been investigated after ESWL; however, available evidence did not demonstrate a significant reduction in SS incidence compared with α-blocker therapy alone [49]. Furthermore, adjunctive α-blocker therapy after ESWL may facilitate fragment passage and improve the expulsion rate of SS, with a potential increase in SFRs compared with conservative management alone [50].

## Discussion

Since its introduction into clinical practice, ESWL has been recognized as a safe and effective non-invasive treatment for kidney stones [51]. Over the years, advancements in shock-wave generator, delivery systems, and imaging guidance have significantly improved stone fragmentation efficacy [52]. Despite these technological developments, SS remains one of the most concerning complications of shock-wave treatment, with a reported incidence that varies widely across studies [53, 54]. As highlighted in this review, a universally accepted definition of SS after ESWL is lacking. The diagnostic classification proposed by Coptcoat et al., which integrates both anatomical findings and management strategies based on symptoms type (asymptomatic, pain only, or pain associated with fever), remains the most reliable and widely validated framework and has been applied in several clinical studies [19]. Nonetheless, the development of SS following ESWL remains unpredictable, being influenced by multiple factors, including shock-wave energy, frequency, stone size, and anatomical location [54–57].

Prophylactic ureteral stenting has been proposed to prevent SS in patients with a large stone burden [58]. However, the presence of a DJ stent often necessitates multiple ESWL sessions and does not appear to significantly improve overall SFR [59, 60]. Although DJ stenting can be performed safely in an outpatient setting [61], current evidence does not support its routine use for treatment outcomes. Indeed, auxiliary procedures are frequently required to achieve complete stone clearance despite the presence of ureteral drainage [62, 63]. Consequently, in cases of large stone burden — typically exceeding 20 mm — PCNL remains the preferred first-line treatment option, whereas FURS (RIRS) should be considered an alternative or second-line approach in selected patients [64].

While SS is generally associated with post-ESWL ureteral obstruction, it may also occur after endourological procedures; however, the lack of detailed characterization has likely led to its under-recognition in clinical practice [65, 66]. In contrast, PCNL may itself represent a cause of this complication, as residual stone fragments not evacuated through the percutaneous tract, as well as small fragments flushed down the ureter by pressurized irrigation, can contribute to SS formation.

Recent technological advances – particularly improvements in laser efficiency, instrument miniaturisation and introduction of FANS – have expanded the role of endourology for treating progressively larger stones. The advent of long-pulse Ho: YAG, TFL and recently p-Tm: YAG has enabled the generation of fine particulate matter, generally referred as “dust”, rather than discrete fragments during FURS [67]. In this setting, dusting techniques are increasingly favoured by endourologists because they reduce the need for basketing and may shorten operative time. However, these approaches have also been associated with a higher likelihood of secondary procedures due to residual debris [68–70]. Although this phenomenon may be mitigated using FANS, a significant proportion of patients still present residual stone particles following endoscopic treatment.

These particles, generally defined as Clinically Insignificant Residual Fragments (CIRFs), typically measures ≤ 4 mm in maximum diameter. They are often considered negligible and, when present, do not usually compromise the definition of SFRs status [70]. Nevertheless, accumulating evidence suggests that CIRFs may have a certain clinical impact, being associated with stone recurrence and postoperative complications [71, 72]. Such residual fragments may pass spontaneously through the ureter; however, they can still cause to ureteral obstruction. When these fragments accumulate, they may contribute to the development of SS. Although NCCT provides excellent diagnostic accuracy, the clinical relevance and accurate characterization of very small fragments may be limited, particularly when fragments are present as fine particulate material rather than discrete calculi. This issue becomes even more relevant when fragments are combined with finer particles ≤ 250 μm, commonly referred to as “dust” [73] — these residuals may aggregate within the urinary tract, resulting in hydronephrosis, pain, or the need for reintervention to achieve complete stone clearance. Although their overall clinical impact remains debated, both CIRFs and micrometric dust have been consistently associated with higher retreatment rates.

The absence of standardized follow-up imaging protocols and concerns regarding cumulative radiation exposure in recurrent stone formers, further limit the accurate detection and estimation of SS incidence after endourological treatments [74, 75]. In addition, the widespread use of US in the emergency department settings reduces diagnostic sensitivity for detecting small or transient ureteral fragments.

In light of these limitations and the absence of a formal clinical definition applicable to contemporary endourological practice, we cautiously propose the descriptive term “*Sandstreet*” in the context of FURS and PCNL. This term is intended to describe the accumulation of CIRFs and stone dust within the ureter — either in the presence or absence of prior drainage — comprising predominant micrometric or small millimetric particles, generally ≤ 4 mm in maximum diameter.

Such accumulations may be asymptomatic and transient, resolving spontaneously, and should therefore be excluded from the definition when clinically insignificant. Nevertheless, the same fragments may result in partial or complete ureteral obstruction with either transient or persistent symptoms. Their clinical relevance can progressively improve thanks to innovative Artificial Intelligence (AI) assisted imaging technologies, which may higher detection rate and characterization of residual fragments [76]. Importantly, this proposed terminology is intended as a descriptive framework rather than a distinct pathological entity, and requires prospective clinical validation under real-world conditions.

**Fig. 3:**
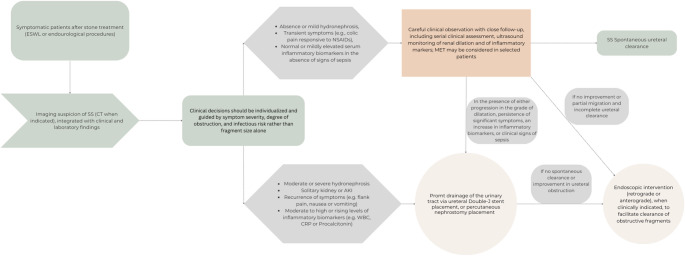
Description of the clinical decision-making process for the management of SS following ESWL or endourological procedures

The management of SS remains a subject of ongoing debate, with therapeutic approaches ranging from conservative management to surgical intervention, depending on the severity of obstruction and the patient’s clinical presentation [46]. While SS typically begins with the accumulation of fragments within the ureter, producing flank pain. If left untreated, persistent obstruction may progress to hydronephrosis, infection and, in severe cases, potentially life-threatening urosepsis [43]. Accordingly, management strategies should be individualized and guided primarily by clinical evolution rather than fragment size alone. Expectant management — including analgesics such as NSAIDs and α-blockers as part of MET — may represent a reasonable option in carefully selected patients to promote spontaneous fragment clearance (Fig. 3.).

The role of ESWL for treating pre-formed SS remains controversial due to its inconsistent efficacy in fragment clearance [39, 43].

In the early postoperative setting, ESWL directed at steinstrasse may improve clearance and shorten time to resolution compared with a conservative approach, potentially avoiding the need for drainage placement [77]. In particular, the presence of a leading fragment > 5 mm has been proposed as a threshold warranting further treatment, given the higher likelihood of persistence and secondary complications [31].

Conversely, patients presenting with acute colic pain, elevated inflammatory markers, or progressive obstruction should undergo prompt decompression with a DJ stent or PNS. URS with laser lithotripsy should be considered the preferred treatment for persistent SS with delayed fragment clearance in non-emergent situations — after adequate infection control — given that reported series have demonstrated SFRs approaching 95% [37, 42, 78]. URS is also indicated when expectant management fails to achieve adequate clearance. However, no consensus currently exists regarding the optimal URS instrumentation, laser settings, or operative techniques to minimize complications [79, 80].

Ureteral mucosal injury — leading to ischemia, fibrosis, and stricture formation — remains a major concern [81]. Particularly attention must be paid to laser energy and frequency parameters during ureteral SS treatment to prevent thermal damage to the ureteral wall. Recently developed flexible aspirating access sheaths, some equipped with real-time intrarenal pressure monitoring, have shown promising results in achieving high SFRs while maintaining procedural safety [41, 42, 82].

In addition, machine learning algorithms have recently been introduced for risk stratification in urolithiasis. These systems hold the potential to enable personalized treatment planning based on patient-specific factors and helping to prevent SS-related complications [83]. Integrating predictive analytics into endourological decision-making could optimize surgical strategies and reduce the occurrence of symptomatic or progressive SS, which continues to represent a clinically significant and sometimes urgent condition.

This study is not without limitations. Considerable heterogeneity among ESWL devices, treatment parameters, laser technologies, and study designs precluded direct comparison and prevented the performance of a meta-analysis. In addition, potential sources of bias and confounding — such as variability in stone composition, operator expertise, and outcome reporting — were not consistently addressed across studies. Consequently, the overall RoB for all the included studies were judged to be moderate to high, which limits the generalizability of the conclusions of the present systematic review. These limitations emphasize the need for a standardized definition of SS, and the development of unified diagnostic and management criteria applicable to both ESWL and endourological procedures. Furthermore, in-vitro tests under high-fidelity conditions, which might comprehend three-dimensional stone and upper tract reproduction, may help to better assess treatment outcomes and procedure-related complications associated with endourological management of SS [84, 85].

Finally, the integration of predictive and imaging-based diagnostic technologies will be crucial to optimize outcomes and reduce both the clinical and economic burden associated with this complication.

## Conclusion

SS remains a well-recognized complication of ESWL, although considerable heterogeneity persists in its definition and management strategies across the literature. Earlier clinical classifications and treatment principles continue to provide a valuable framework for patient care. Nevertheless, advances in endoscopic technology and laser lithotripsy have broadened the therapeutic scope for large stone burdens, while simultaneously introducing new challenges.

The recognition of this emerging entity — termed “Sandstreet” — in the context of endourological interventions, together with a comprehensive understanding of preoperative and patient-specific factors, represents a crucial step toward improving prevention, diagnosis, and management of SS-like conditions.

## Supplementary Information

Below is the link to the electronic supplementary material.

Supplementary Material 1

## Data Availability

No datasets were generated or analysed during the current study. The corresponding author has full access to all the data in the study and takes responsibility for the integrity of the data and the accuracy of the analysis.

## Abbreviations

SS: Steinstrasse
ESWL: Extracorporeal shock wave lithotripsy
URS: Retrograde ureterorenoscopy
FURS: Flexible ureterorenoscopy
FANS: Flexible aspirating navigable urteral access sheaths
PCNL: Percutaneous nephrolithotomy
Ho:YAG: Holmium:yttrium-aluminum-garnet (laser)
TFL: Thulium fiber laser
SFR: Stone-free rate
DJ: Double-J ureteral stent
CIRFs: Clinically insignificant residual fragments
MET: Medical expulsive therapy
NSAIDs: Non-steroidal anti-inflammatory drugs
KUB: Plain radiography
NCCT: Non-contrast computed tomography
US: Ultrasound
HU: Hounsfield units
RoB: Risk of bias
RCT: Randomized controlled trial
PNS: Percutaneous nephrostomy
EAU: European association of urology
PRISMA: Preferred reporting items for systematic reviews and meta-analyses
PROSPERO: International prospective register of ongoing systematic reviews
