# Supplementary material for: Understanding steinstrasse: a systematic review of definitions, clinical management, and emerging perspectives in endourology
Source: World J Urol. 2026 Feb 16;44(1):171. doi: 10.1007/s00345-026-06267-7 (PMC12909395; doi:10.1007/s00345-026-06267-7)
Supplement: Supplementary file 1 — Supplementary Material 1 [file 345_2026_6267_MOESM1_ESM.docx]

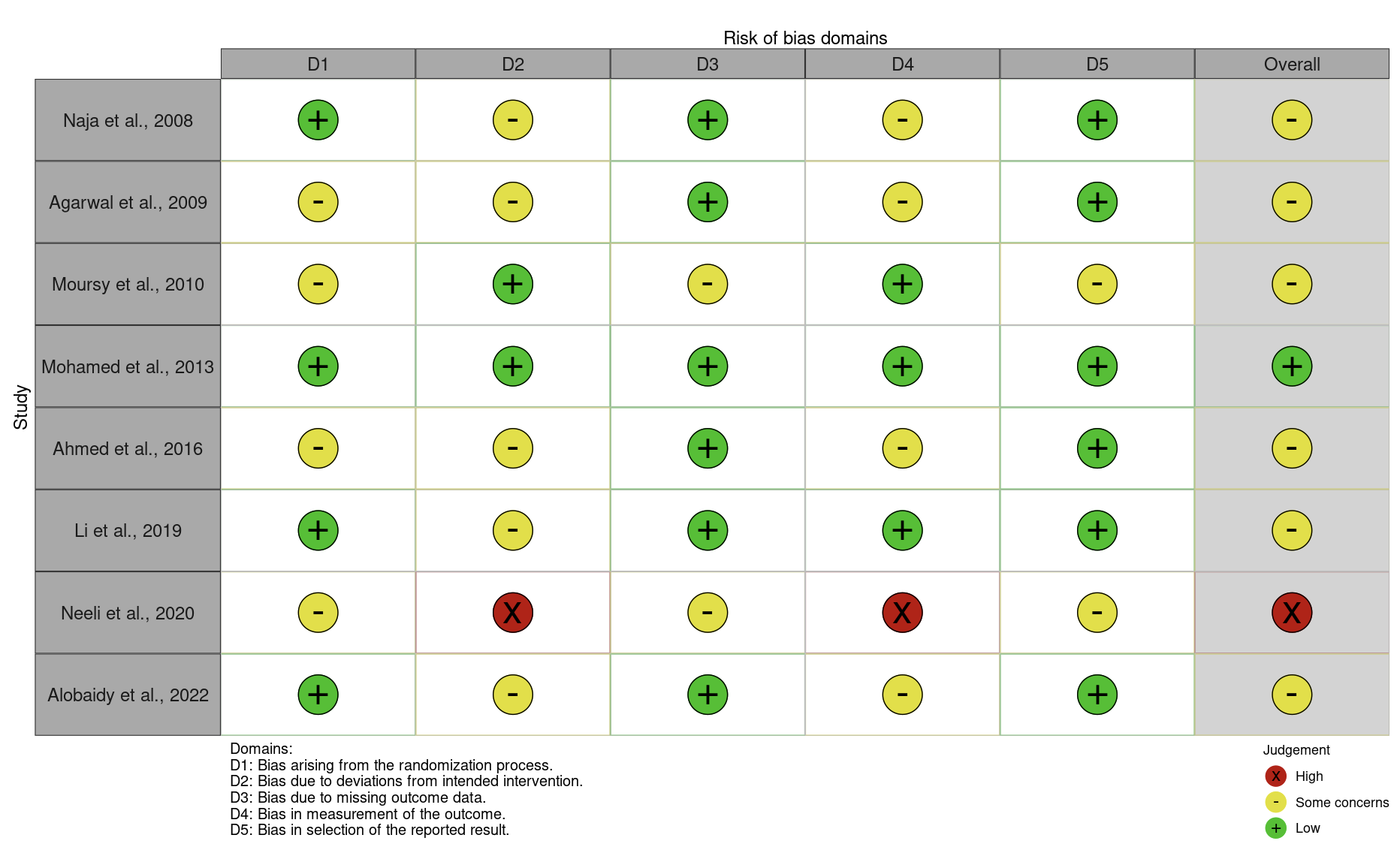
**Supplementary Table 1.** Risk of Bias assessment of randomized studies of interventions


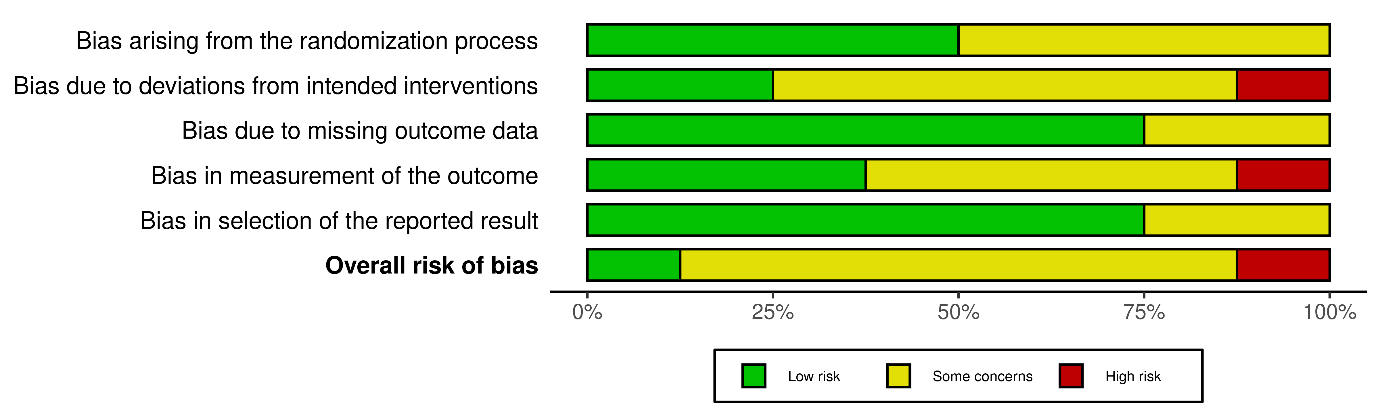


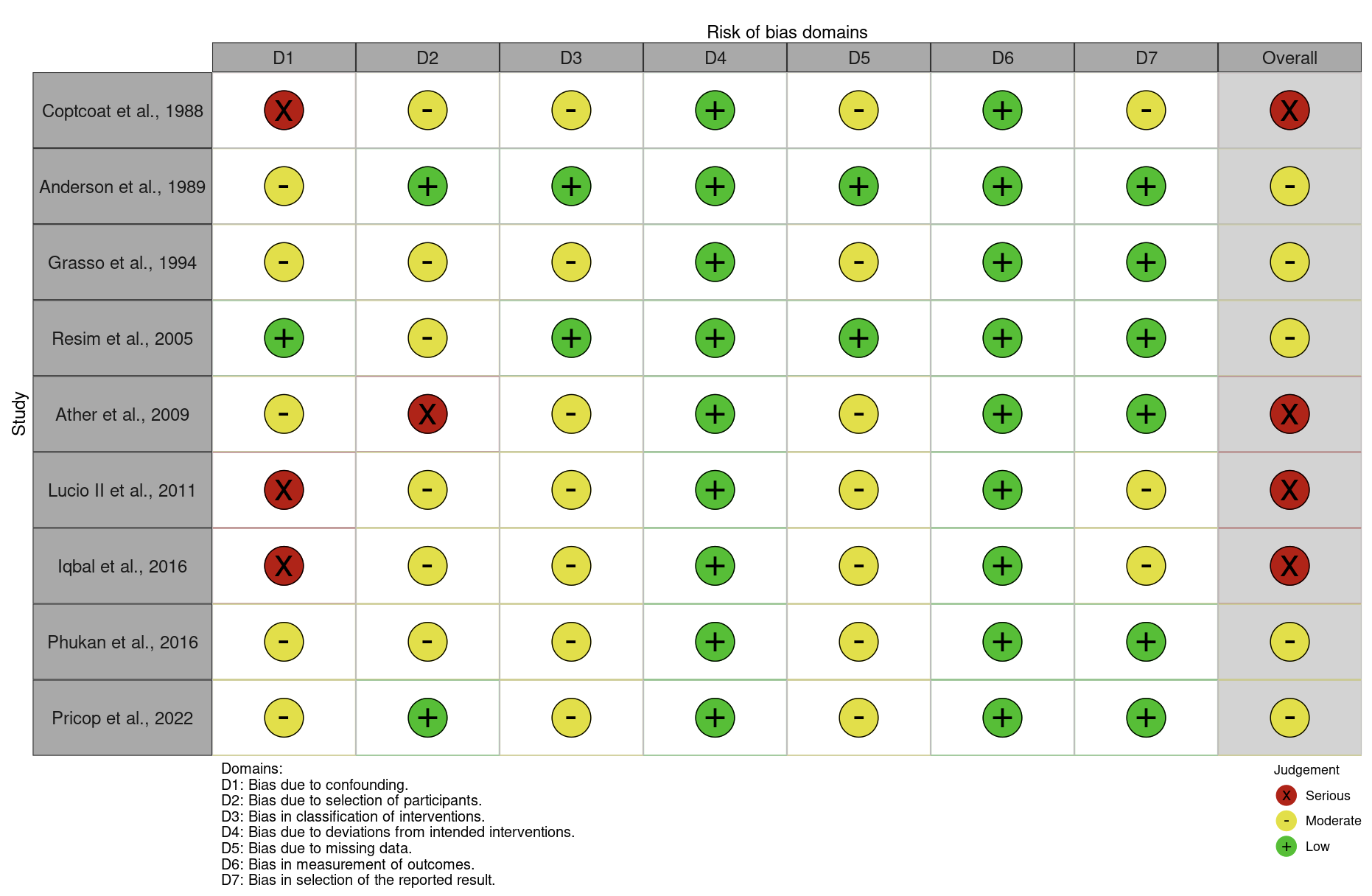
**Supplementary Table 2.** Risk of Bias assessment of non-randomized studies of interventions


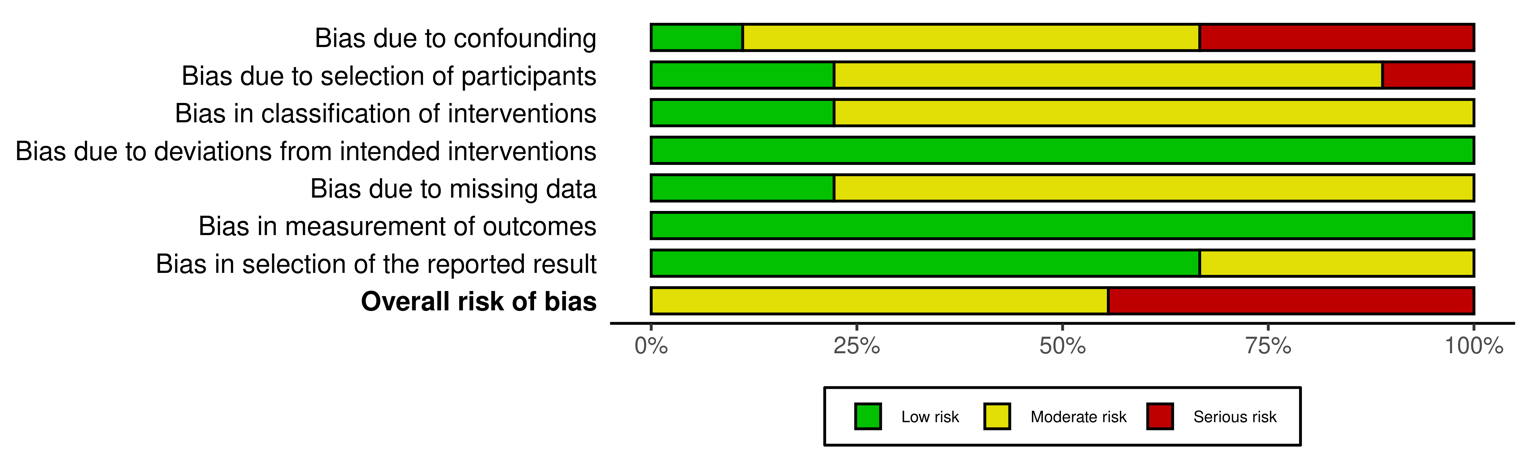


**Supplementary Table 3.** Risk of Bias assessment of single-arm studies

| **Author, Year** | **Was there an a priori protocol?** | **Was the total population included or were study participants selected consecutively?** | **Was outcome data complete for all participants and any missing data adequately explained/unlikely to be related to the outcome?** | **Were all prespecified outcomes of interest and expected outcomes reported?** | **Were primary benefit and harm outcomes appropriately measured?** | **RoB low/high** |
| --- | --- | --- | --- | --- | --- | --- |
| Coptcoat et al., 1986 | No | Yes | No | Yes | Yes | High |
| Chaussy et al., 1987 | No | No | No | Yes | Yes | High |
| Böhle et al., 1989 | No | No | No | Yes | Yes | High |
| Fine et al., 1989 | No | No | No | No | No | High |
| Weinerth et al., 1989 | No | No | Yes | Yes | Yes | High |
| Kim et al., 1991 | No | No | No | Yes | Yes | High |
| Küpeli et al., 1998 | No | No | No | Yes | Yes | High |
| Sulaiman et al., 1999 | No | No | No | Yes | Yes | High |
| Sayed et al., 2001 | No | Yes | Yes | Yes | Yes | Low |
| Madbouly et al., 2002 | No | No | No | Yes | Yes | High |
| Soyupek et al., 2005 | No | No | No | Yes | Yes | High |
| Goyal et al., 2006 | No | No | No | Yes | Yes | High |
| Yoshida et al., 2007 | No | Yes | Yes | No | Yes | Low |
| Rabbani et al., 2008 | No | No | No | No | No | High |
| Shrestha et al., 2010 | No | Yes | Yes | Yes | Yes | Low |
| Al-Marhoon et al., 2013 | No | Yes | Yes | Yes | Yes | Low |
| Feng et al.,2013 | No | No | Yes | Yes | Yes | High |
| El‑Assmy et al., 2015 | No | No | Yes | Yes | Yes | High |
| Kang et al., 2017 | No | Yes | Yes | Yes | Yes | Low |
| Dobrowiecka et al., 2018 | No | No | No | Yes | Yes | High |
| Parmar et al., 2021 | Yes | Yes | No | Yes | Yes | High |
| Ivanov et al., 2022 | Yes | Yes | Yes | Yes | Yes | High |
| Wang et al., 2023 | No | Yes | Yes | Yes | Yes | High |
| Elbaset et al., 2024 | No | Yes | Yes | Yes | Yes | High |
| Yuming et al., 2024 | No | Yes | Yes | Yes | Yes | High |

**Supplementary table 4.** Classification of SS based on clinical management proposed by Coptcoat et al. (1988), categorized according to clinical presentation, imaging findings, and recommended therapeutic strategy.

|  | **Clinical presentation** | **Proposed treatment** |
| --- | --- | --- |
| **Group 1** | Asymptomatic SS | Managed conservatively with weekly radiological and ultrasound examinations until spontaneous passage of fragments |
| **Group 2** | Symptomatic SS without proximal dilatation | Single-dose Intramuscular Gentamicin, followed by oral trimethoprim and analgesia until spontaneous passage of stone fragments |
| **Group 3** | Symptomatic SS with proximal dilatation or fever | PNS under local anesthesia. If no spontaneous clearance occurs after 3 weeks, a combined endoscopic approach (PCNL and URS) is recommended. Failure of endoscopic management is an indication for open ureterolithotomy |

**Abbreviations**: SS = Steinstrasse; URS = retrograde ureterorenoscopy; PCNL = percutaneous nephrolithotomy; PNS = percutaneous nephrostomy.

**Supplementary Table 5**: Studies analyzing predictive features, prevention strategies, and therapeutic interventions for SS following ESWL and endourological procedures

| **Authors**  **(year)** | **Study design** | **Number of patients** | **Study aim** | **Procedure/ lithotripsy settings** | **Stone location**  **and dimension** | **Steinstrasse Definition** | **Incidence and localization of Steinstrasse** | **Treatment purposed** | **Predictive features** |
| --- | --- | --- | --- | --- | --- | --- | --- | --- | --- |
| Anderson et al (1989) | Retrospective,  Adults | 36 patients (46 renal units) | To evaluate the management of large renal calculi treated with PNS + ESWL, or Stent + ESWL, or ESWL alone | Electrohidraulic, lithotripsy (18-24 kV | Kidney stones ≥3 cm | Retained ureteral fragments causing ureteral obstruction | SS occurred in 1/12 (8%) after PNS + ESWL,  7/21 (33%) after DJ stent + ESWL,  5/8 (62%) after ESWL alone | Expectant management, URS with basket retrieval, DJ or PNS placement for symptomatic obstruction | ND |
| Fine et al.  (1989) | Retrospective, Adults | 1000 patients | To evaluate the function of ureteral stents during voiding cystography   \|  \| \| --- \| | ND | ND | Obstruction of the ureter by sand/fragment boluses following ESWL of large renal stones | ND | 170 prophylactic stents placed prior to ESWL for stones ≥ 2cm under fluoroscopic guidance | Stents prevent SS formation and facilitate faster fragment clearance |
| Kim et al.  (1991) | Retrospective, Adults | 958 | To evaluate repeat ESWL for complicated SS | Piezoelectric lithotripter | Renal pelvis (56,4%),  Calyces (32.7%),  UPJ (10.9%) | Arrest of spontaneous fragment passage through ureter | 55/958 (5,7%):  distal ureter 60%,  proximal ureter 32.7%,  mid ureter 7.3% | Expectant management (n=35, 63.6%),  repeat ESWL (n=18, 32,8%),  URS (n= 1), open ureterolithotomy (n= 1) | Repeat ESWL effective for SS management |
| Sulaiman et al  (1999) | Retrospective,  Adults | 1087 | To compare outcomes between pre-stented and non-stented patients | Piezoelectric lithotripter; 75 shock waves/ minute | Stone 10 - 95 mm | Accumulation > 2 fragments in any ureteral segment | 69/ 1087 (6,3%):  distal ureter 72%, proximal 18%,  middle 10% | Expectancy management (46,4%),  Endoscopy (39.1%),  ESWL on SS (14,5%) | ND |
| Sayed M. A. B. et al (2001) | Retrospective,  Adults | 885 patients | To determine causes, methods of prevention, treatment strategies | Eltrohydraulic lithotripsy,17-26 kV | Upper tract stones <3 cm | Gravel-like fragments migrating together and obstructing the ureter post- ESWL | 52 / 885 (5.9%); Distal 64%, proximal 29%, mid 8% | Expectant (48%),  repeated ESWL (23%),  PNS (19%), URS (6%), open surgery (4%) | Active treatment required for obstruction/infection.  ESWL and PNS most effective; URS and open surgery reserved for complex cases |
| Kupeli B. et al  (2003) | Retrospective, Adults | 6300 | To evaluate outcomes of SS after ESWL | Electromagnetic lithotripter;  mean 17.8 kV, 2900 shock waves/treatment | Stone size varied 4-42 mm, ≥21 mm stented | Multiple ureteral fragments after ESWL | 360 / 6300 (5.7%) | Expectant, ESWL on SS, URS, ureterolithotomy | Endoscopic treatment preferred for stones > 3 cm^2^ |
| Ather M. H. et al (2008) | Retrospective, Adult + children | 4644 | To assess factors affecting SS and impact of pre-ESWL stenting | Electrohydraulic lithotripter | Mean size 16.8mm | Dynamic SS = stones in transit; Static SS = as a persistent SS on ≥ 2 consecutive imaging studies ≥ 1 week apart. | 326 /4644 (7%),  SS localized in distal ureter in 89%, middle ureter in 9%, proximal 2% | Expectant (54%), intervention (46%): URS, ESWL on SS, PNS, open ureterolithotomy. | Pre-ESWL DJ stenting reduce acute SS, but not overall need for intervention |
| Iqbal N. et al  (2016) | Retrospective,  Adult | 200 | To compare outcomes of URS vs ESWL for proximal ureteral stones | ND | Proximal radiopaque single stone < 2cm | Impacted fragments causing ureteral obstruction | 4 / 200 (2%) after ESWL | ND | ND |
| Parmar K. et al  (2021) | Prospective Observational, Adult | 684 | To assess the aetiology and management issues of large spontaneous SS | ND | Renal/ureteral stones post-ESWL | Multiple ureteral stones, often asymptomatic, but potentially obstructive causing loss of renal function | 28 / 684 (4.1%): 19 (2.8%) post- ESWL, 9 (1.31%) spontaneous SS (1 bilateral) | Spontaneous SS: 5 ureterolithotomy, 2 PCNL, 1 URS, and 1 nephroureterectomy | Prompt treatment essential to prevent renal loss; metabolic evaluation mandatory |
| Ivanov A. S.  (2022) | Retrospective,  Adult | 76 | To assess efficacy of WiScope® single-use flexible ureterorenoscope | FURS + 12/14Fr UAS; 273micron Ho:YAG laser (1 J, 10 - 20 Hz) | mean size 1.34;  upper calyx 16 cases, middle calyx in 20, lower calyx in 18,  pelvis in 22 | Ureteral fragment aggregation causing obstruction | 1 / 76 (1.3%),  distal ureter | Success 72 / 76 (94.7%), SFR of 92.1%,  lower calyx 77.7%,  middle-upper 96.5% | ND |

**Abbreviations**: SS = Steinstrasse; FURS= flexible ureteroscopy; ESWL = extracorporeal shock-wave lithotripsy; URS = retrograde ureterorenoscopy; PCNL = percutaneous nephrolithotomy; PNS = percutaneous nephrostomy; DJ = Double-J stent; FANS = Flexible Aspirating Navigable Ureteral Access Sheath ; Ho:YAG = Holmium:Yttrium–Aluminum–Garnet laser

**Supplementary Table 6:** Randomized controlled trials and retrospective studies evaluating the efficacy of medical expulsive therapy for the clearance of SS.

| **Authors (year)** | **Study design** | **Study aim** | **Population / treatment groups** | **Steinstrasse Definition** | **Incidence of SS** | **Localization & dimensions** | **Stone clearance** |
| --- | --- | --- | --- | --- | --- | --- | --- |
| Resim et al.  (2005) | RCT,  Adults | To evaluate the efficacy of tamsulosin in lower-ureter SS clearance | 436 patients treated with ESWL, 67 developed SS and were randomized:  Group 1(n= 35) NSAID + expectant management,  Group 2 (n =32) NSAID + Tamsulosin daily | Column of stone fragments developing within the ureter after SWL | 67 patients with SS in the lower ureter | Distal third of the ureter | SS resolved in 23/35 (65.7%) of Group 1 and 24/32 (75%) of Group 2 at 6-week follow-up |
| Naja et al.  (2005) | RCT, Adults | To evaluate alpha-blocker efficacy on SS resolution in the lower ureter | 139 patients with single renal lithiasis underwent weekly ESWL ≤ 3 months;  Group 1 (n= 51) tamsulosin, Group 2 (n= 65) analgesic only | Column of retained fragments in the ureter associated with pain | 11 patients developed SS (2 in group1, 9 in group 2) | ND | ND |
| Agarwal et al. (2009) | RCT,  Adults | To assess efficacy of MET for upper-tract ureteral calculi | 40 patients with single upper-ureteral stone  treated with ESWL;  Group 1 (n= 20) tamsulosin; Group 2 (n = 20) no MET | Column of retained fragments associated with pain | 9 patients (22.5%) developed SS | ND | SFR 95% in group 1 vs 90% in group 2 |
| Moursy et al. (2010) | RCT,  Adults | To evaluate efficacy of tamsulosin for SS clearance | 1564 patients scheduled for ESWL;  88 with unilateral SS randomized: Group 1 (tamsulosin) vs group 2 (expectant). | Column of retained fragments obstructing the pelvicalyceal system | 88 patients with SS | Mean SS length: 6 cm (group 1), 6.25 cm ( group 2) | Stone expulsion: 32/44 (72.7%) in Group 1, 25/44 (56.8%) in group 2 |
| Mohamed et al.(2013) | RCT,  Adults | To compare stone passage rate and pain after ESWL ± tamsulosin | 130 patients with solitary ureteric stone treated by ESWL; group 1 (n= 65) tamsulosin; group 2 (n= 65) analgesics only | Column of fragments in the ureter following ESWL | 6/130 (4,.6%) developed SS (4 in group 1, 2 in group 2) | Distal ureter | SFR 85% in group 1; 89% in group 2 |
| Ahmed et al.  (2016) | RCT,  Adults | To evaluate adjunctive tamsulosin therapy after ESWL on renal stones | 249 patients with solitary renal stones; Group 1 (n = 123) tamsulosin + analgesia; Group 2 (n = 126) analgesia only (≤ 12 weeks) | SS = simultaneous mobilization of multiple fragments lodging in ureter causing obstruction and colic | 14/249 (5.6%): 5 group 1 (4.1%), 9 group 2 (7.1%) | ND | 6/9 SS in group 2 required urgent intervention (PNS or DJ). SFR 78% group 1 vs 69% group 2 |
| Li et al.  (2020) | RCT,  Adults | To assess whether sexual intercourse aids stone clearance after ESWL for distal-ureteral calculi (7-15 mm) | 209 patients randomized:  Group 1 (n= 70) sexual intercourse ≥ 3 x/week;  Group 2 (n= 71) tamsulosin daily;  Group 3 (n= 68) control therapy | Presence of > 1 ipsilateral ureteral stone after SWL | SS developed in 2/70 (2.9%) group 1; 2/71 group 2 (2.8%); 8/68 group 3 (11.8%). | Distal ureter | SFR after 2 weeks: 80% group 1; 81.7% group 2; 63.2% group 3. |
| Neeli et al. (2021) | RCT,  Adults | to compare tamsulosin alone vs tamsulosin + tadalafil for post-ESWL clearance | 140 patients with solitary renal calculi < 20 mm (non-lower calyx); Group 1 (n= 74) tamsulosin; group 2 (n =74) tamsulosin + Tadalafil | Column of fragments retained in the ureter with pain | SS in 20 (13,5%);  13 (17,6%) group 1, 7 (9,6%) group 2 | SS length ≤12mm in 4 cases; > 12 mm in 16 cases) | SFR 72.5% in group 1; 90% in group 2 |
| Goyal et al.  (2023) | Retrospective,  Adults & Children | 1000 patients with urinary stones treated by ESWL | To assess outcome of expectant management according to SS type | Recognized ESWL complication influenced by stone size, location, composition, energy settings. | SS occurred 60/1000 patients (6%) after ESWL (<1 cm = 0.6%; 1–2 cm = 6.1%; 2–3 cm = 11.1%; 3–4 cm = 18.5%) | Distal ureter 53%, upper 33%, mid 14%;  SS length 1-9cm | Expectant management sucess 50%; re-ESWL 45%; URS required in 5% |
| Elbaset et al.  (2024) | Retrospective,  Adults | 145 patients with SS after ESWL; all received tamsulosin daily ± re-ESWL if needed | To define predictors of MET success after ESWL for uncomplicated SS | Aggregation of fragments in ureter post- ESWL | 145 with SS | ND | SFR 40/145 (27.6%) with only tamsulosin; with tamsulosin + re-ESWL 48%; 26/ 145 (17.9%) required urgent surgery dor complications |

**Abbreviations**: URS = Ureterorenoscopy, PNS = Percutaneous Nephrostomy, NSAIDs = Non-Steroidal Anti-Inflammatory Drugs, ESWL= Extracorporeal Shock Wave Lithotripsy, re-ESWL= repeat session(s) of ESWL, RCT = Randomized Controlled Trial, SS = Steinstrasse; US = Ultrasound, SFR = Stone Free Rate

**Appendix 1:** Detailed search strategy across database.

**Common Core across database**

("steinstrasse" OR "sandstrasse" OR "stein street" OR "stone street")

AND

("urolithiasis" OR "kidney stones" OR "renal calculi" OR "ureteral calculi" OR "urinary calculi" OR "stones" OR "calculi").

**PubMed**

("steinstrasse"[All Fields] OR "sandstrasse"[All Fields] OR "stein street"[All Fields] OR "stone street"[All Fields])

AND

("urolithiasis"[MeSH Terms] OR "urolithiasis"[All Fields] OR "kidney stones"[All Fields] OR "renal calculi"[All Fields] OR "ureteral calculi"[All Fields] OR "urinary calculi"[All Fields] OR "stones"[All Fields] OR "calculi"[All Fields]).

**Embase**

('steinstrasse' OR 'sandstrasse' OR 'stein street' OR 'stone street')

AND ('urolithiasis'/exp OR 'kidney stone' OR 'renal calculi' OR 'ureteral calculi' OR 'urinary calculi' OR 'stones' OR 'calculi').

**Scopus**

("steinstrasse" OR "sandstrasse" OR "stein street" OR "stone street")

AND

("urolithiasis" OR "kidney stones" OR "renal calculi" OR "ureteral calculi" OR "urinary calculi" OR "stones" OR "calculi").
